# CpG ODN Activates TLR9 and Upregulates TLR3 via the p38 MAPK-ATF3 Signaling Axis to Synergistically Enhance Dendritic Cell Vaccine Efficacy

**DOI:** 10.3390/cells14221785

**Published:** 2025-11-13

**Authors:** Lv Zhou, Zhuowei Lei, Qian Jiang, Linpeng Xu, Quanji Wang, Yimin Huang, Ting Lei

**Affiliations:** 1 Sino-German Neuro-Oncology Molecular Laboratory, Department of Neurosurgery, Tongji Hospital, Tongji Medical College, Huazhong University of Science and Technology, Jiefang Avenue 1095, Wuhan 430030, China; m202376657@hust.edu.cn (L.Z.); tjlzw2018@tjh.tjmu.edu.cn (Z.L.); d202382335@hust.edu.cn (Q.J.); xulinpeng9268@163.com (L.X.); quanjiwang@tjh.tjmu.edu.cn (Q.W.); 2 Hubei Key Laboratory of Neural Injury and Functional Reconstruction, Huazhong University of Science and Technology, Wuhan 430030, China; 3 Department of Orthopedics, Tongji Hospital, Tongji Medical College, Huazhong University of Science and Technology, Jiefang Avenue 1095, Wuhan 430030, China

**Keywords:** immunotherapy, dendritic cell vaccine, glioma, toll-like receptor

## Abstract

**Highlights:**

**What are the main findings?**

- The synergistic application of CpG ODN and Poly(I:C) enhances the antitumor efficacy of dendritic cell vaccines derived from mouse bone marrow.
- The underlying mechanism for this enhanced effect may be associated with the p38 MAPK-ATF3 signaling axis.

**What are the implications of the main findings?**

- Combinatorial administration of these agonists can be utilized to engineer dendritic cell vaccines with improved functionality.
- Employing a sequential activation strategy can further augment this antitumor effect.

**Abstract:**

Toll-like receptor 9 (TLR9) and Toll-like receptor 3 (TLR3), which are widely expressed in dendritic cells (DCs), function as key pattern recognition receptors (PRRs) in the immune system. Their primary roles involve specifically detecting pathogen-associated molecular patterns (PAMPs): TLR9 recognizes unmethylated CpG motifs predominantly found in bacterial and viral DNA, while TLR3 identifies viral double-stranded RNA (dsRNA), a molecular signature associated with viral replication. Their specific agonists [CpG ODN (a TLR9 agonist) and poly(I:C) (a TLR3 agonist)] can effectively activate DCs and enhance the expression of immune activation-related molecules. In this study, by establishing a mouse primary dendritic cell model and a glioma-bearing mouse model, and employing techniques such as transcriptome sequencing, we found that combined stimulation with CpG ODN and poly(I:C) significantly enhanced the anti-tumor function of DCs: in vitro, DCs subjected to combined stimulation showed upregulation of anti-tumor-related surface markers, enhanced migratory capacity, and a more effective activation of CD8^+^ T cells; in vivo, a DC vaccine loaded with tumor lysate antigen and stimulated with this combined regimen significantly delayed the progression of glioma in tumor-bearing mice. Further investigation revealed that the underlying mechanism for this enhanced effect may involve TLR9 activation promoting TLR3 upregulation through the p38 MAPK-ATF3 signaling axis. Consequently, we designed a sequential stimulation protocol (first CpG ODN then poly(I:C)), which demonstrated a stronger anti-glioma effect compared to simple combined stimulation. This study provides a new strategy for enhancing the immune efficacy of DC vaccines and has potential significance for promoting the clinical translation of DC vaccines.

## 1. Introduction

Patients with World Health Organization (WHO) grade III–IV central nervous system (CNS) gliomas exhibit a median overall survival of approximately 12–18 months following conventional surgical intervention [1]. Owing to the limited sensitivity of these tumors to radiotherapy and chemotherapy [2,3], novel therapeutic modalities such as immunotherapy [4], cellular therapy, and oncolytic virotherapy have been extensively explored in preclinical research [5,6]. Dendritic cell (DC) vaccination represents a promising immunotherapeutic strategy [7]. This approach involves the differentiation of autologous peripheral blood-derived monocytes into DCs, which are subsequently pulsed with tumor lysate antigens ex vivo and reinfused into the patient. As professional antigen-presenting cells (APCs), DCs process and present the loaded antigens to CD8^+^ and CD4^+^ T cells within lymphoid organs, thereby indirectly eliciting antitumor immune responses [8,9]. However, similar to other emerging therapies, DC-based vaccines face significant challenges, including immune evasion mechanisms and the highly immunosuppressive tumor microenvironment (TME) characteristic of CNS gliomas [10]. Although clinical trials of DC vaccines for glioma patients are underway, the autologous nature of these vaccines presents inherent limitations: DCs are often terminally differentiated and exhibit poor antigen-presenting capacity, insufficient T cell activation, and impaired migratory ability [11,12,13,14,15]. Furthermore, the complex and costly procedures for DC extraction and induction, coupled with their suboptimal biological functionality, impose substantial physiological and economic burdens on patients. Toll-like receptors (TLRs) play pivotal roles in innate immunity. Previous studies have reported that activation of TLR3 and TLR9 can enhance the expression of antitumor surface markers on DCs [16,17,18,19]. Members of our research group have previously reported that combined activation of TLR3 and TLR9 may enhance the antitumor function of microglia through a synergistic effect, thereby offering a new strategy for glioma immunotherapy [20]. Since both dendritic cells and macrophages are professional antigen-presenting cells, we hypothesize that co-activation of TLR3 and TLR9 can potentiate the antitumor efficacy of dendritic cell vaccines. This study aims to develop a cost-effective strategy to augment the antitumor efficacy of DC vaccines and to elucidate the underlying mechanisms.

Using a murine glioma model, we investigated a modified DC vaccine strategy involving the synergistic ex vivo activation of TLR9 and TLR3 with CpG ODN and poly(I:C), respectively. This combined stimulation enhanced DC migration, antigen presentation, and CD8^+^ T cell activation capacity. In vivo experiments demonstrated that this approach significantly delayed tumor progression and extended the survival of glioma-bearing mice. Integrated transcriptomic sequencing and Western blot analyses revealed that the synergistic mechanism may involve TLR9-mediated upregulation of TLR3 expression. Further experiments indicated that sequential administration of the agonists (TLR9 activation followed by TLR3 activation) yielded more pronounced enhancements in DC function and antitumor efficacy, likely due to the time-dependent receptor upregulation requirement.

This study provides a novel strategy for improving the efficacy of human-derived DC vaccines and offers preliminary insights into the associated mechanistic pathways.

## 2. Materials and Methods

### 2.1. Ethical Approval

The study protocol was reviewed and approved by the Animal Ethics Committee (TJH-24-07-046, Approval Date: 19 December 2024) and the Clinical Ethics Committee (TJ-IRB20220325, Approval Date: 7 September 2022) of Tongji Hospital, Huazhong University of Science and Technology. Written informed consent was obtained from both volunteers who provided blood samples.

### 2.2. Cell Culture and Processing

The mouse glioma cell lines (GL261 and CT2A) and human glioma cell lines (LN229 and U251) used in this study were obtained from the American Type Culture Collection (ATCC, Manassas, VA, USA) and confirmed to be free of mycoplasma contamination. All glioma cell lines were maintained in high-glucose DMEM medium (G4612, ServiceBio, Wuhan, China) supplemented with 10% fetal bovine serum (FBS, A5661701, Gibco, Plainville, MA, USA) and 1% penicillin-streptomycin solution (G4003, ServiceBio, Wuhan, China) at 37 °C in a humidified incubator. The culture medium was replaced every 24–48 h. When cells reached 70–80% confluence as observed under an optical microscope (Olympus, Tokyo, Japan), they were either subjected to experimental procedures or passaged/frozen using trypsin digestion solution (G4001, ServiceBio, Wuhan, China). Mouse-derived dendritic cells (DCs) were isolated from the bone marrow of femurs of 6–10-week-old male C57BL/6 mice. These cells were induced to differentiate in RPMI-1640 medium (G4531, ServiceBio, Wuhan, China) containing recombinant GM-CSF (HY-P7361, MCE, Shanghai, China) and IL-4 (HY-P70644, MCE, Shanghai, China) for 7 days before experimental use. Mouse-derived lymphocytes were extracted from the spleens of 6–10-week-old male C57BL/6 mice using lymphocyte separation medium (DKW33, DAKEWE, Shenzhen, China) and cultured in RPMI-1640 medium. Human CD8^+^ T cells were obtained from the same two volunteers who provided peripheral blood samples for human dendritic cell isolation. CD8^+^ T cells were isolated from peripheral blood and used for co-culture experiments.

### 2.3. Isolation of Human CD8^+^ T Cells

Human CD8^+^ T cells were isolated from heparinized peripheral blood of healthy volunteers. Briefly, the blood sample was diluted with a 10-fold volume of cold isolation buffer. Cell isolation was then carried out using the Dynabeads™ Untouched™ Human CD8^+^ T Cells Kit (11348D, Thermo Fisher, Waltham, MA, USA) according to the manufacturer’s protocol, which is based on a negative selection principle to obtain untouched, high-purity CD8^+^ T cells.

### 2.4. Animals

C57BL/6 mice (6–10 weeks old) used in this study were obtained from Hubei BIONt Biological Technology Co., Ltd. (Wuhan, China). The mice were housed under specific pathogen-free (SPF) conditions in the animal facility of the Research Building at Tongji Hospital.

### 2.5. Preparation of Dendritic Cell Vaccines

Tumor lysate-derived whole antigen was prepared from glioma cell lines. Briefly, the harvested glioma cells were subjected to five cycles of rapid freezing in liquid nitrogen for 3 min followed by immediate thawing in a water bath at 37 °C for 2 min. The resulting lysate was then filtered through a 40 um cell strainer to obtain the whole antigen preparation. For mouse bone marrow-derived dendritic cells (BMDCs), the whole antigen derived from GL261 or CT2A glioma cell lines was added to the dendritic cell culture medium at a ratio of 2:1 (glioma cells to dendritic cells) and incubated for 24 h to allow antigen uptake. For human engineered dendritic cells, the whole antigen prepared from LN229 or U251 glioma cell lines was loaded at a ratio of 4:1 (glioma cells to dendritic cells) under the same incubation conditions. The antigen-loaded dendritic cells were then used as dendritic cell vaccines.

### 2.6. Co-Culture System

#### 2.6.1. CFSE Labeling of CD8^+^ T Cells

Mouse splenic lymphocytes or human peripheral blood-derived CD8^+^ T cells were pre-labeled with CFSE dye (E-CK-A345, Elabscience, Wuhan, China) at a concentration of 5 uM for 20 min at 37 °C protected from light. After incubation, the staining was quenched by adding a five-fold volume of complete culture medium (RPMI 1640 supplemented with 10% FBS). The cells were then washed thoroughly three times with phosphate-buffered saline (PBS) to remove any residual unbound dye and resuspended in fresh complete medium. Prior to formal experiments, the proportion and purity of CD8^+^ T cells were confirmed by flow cytometric analysis using anti-CD3 (300311, 155606, DAKEWE, Shenzhen, China) and anti-CD8 (100707, 344706, DAKEWE, Shenzhen, China) antibodies.

#### 2.6.2. Preparation of Dendritic Cell (DC) Vaccines

Mouse Bone Marrow-Derived DC Vaccines: Mouse bone marrow-derived dendritic cells (BMDCs) were stimulated for 24 h under one of the following four conditions: (1) PBS (untreated control); (2) 10 ug/mL poly(I:C) alone; (3) 10 ug/mL CpG ODN alone; (4) a combination of 10 ug/mL poly(I:C) and 10 ug/mL CpG ODN. Following stimulation, the DCs were pulsed with whole tumor lysate antigen (at a DC to tumor cell ratio of 1:3) for an additional 24 h to generate antigen-loaded DC vaccines.

Human Engineered DC Vaccines: Human engineered dendritic cells were subjected to one of four distinct activation protocols: (1) PBS treatment for 24 h (control); (2) simultaneous treatment with 10 ug/mL poly(I:C) and 15 ug/mL CpG ODN for 24 h; (3) simultaneous treatment with 15 ug/mL poly(I:C) and 10 ug/mL CpG ODN for 24 h; (4) sequential treatment involving pretreatment with 10 ug/mL CpG ODN for 24 h followed by 10 ug/mL poly(I:C) for another 24 h. Subsequently, all groups were loaded with whole tumor lysate antigen (at a DC to tumor cell ratio of 1:6) for 24 h to form the final DC vaccines.

#### 2.6.3. Co-Culture and Sample Collection

Both mouse and human antigen-loaded DC vaccines were harvested, washed extensively with PBS to remove residual culture components, and counted. The washed DC vaccines were then co-cultured with the pre-prepared CFSE-labeled CD8^+^ T cells at an effector-to-target ratio of 4:1 (CD8^+^ T cells to DCs) in RPMI 1640 medium supplemented with 10% fetal bovine serum (FBS) and 1% penicillin-streptomycin (dual antibiotics). The co-culture system was maintained in a humidified incubator at 37 °C with 5% CO_2_ for 48 h. After the incubation period, the co-culture supernatant was carefully collected for subsequent cytokine analysis by ELISA, while the cell pellets were harvested for flow cytometric analysis of T cell proliferation (based on CFSE dilution) and activation markers. The measurements for all parameters within the co-culture system were conducted in three biologically independent replicates.

### 2.7. Cytotoxicity Assay of CD8^+^ T Cells

The in vitro CD8^+^ T cell cytotoxicity was evaluated using a co-culture system comprising CD8^+^ T cells, dendritic cells (DCs), and luciferase-transfected tumor cells. Following 48 h of co-culture, the cells were lysed, and potassium luciferin substrate was added to the system. The bioluminescence intensity, which inversely correlates with the number of viable tumor cells, was measured using a multifunctional microplate reader within 5–25 min after substrate addition.

### 2.8. Engineered Dendritic Cells

The human-derived engineered dendritic cell (DC) vaccine was developed and provided by Beijing Celarts Biosciences Group Co., Ltd. (Beijing, China). Peripheral blood samples, used for the induction of dendritic cells and the isolation of CD8^+^ T cells, were obtained from two healthy adult volunteers recruited at Tongji Hospital, Huazhong University of Science and Technology.

### 2.9. Brain Tissue Sectioning and Staining

When glioma-bearing mice exhibited clear tumor-related symptoms such as hemiplegia, convulsions, and significant weight loss, they were administered muscle relaxants and an overdose of anesthetic. The limbs were secured, and transcardial perfusion was performed using phosphate-buffered saline (PBS). Subsequently, the brain tissue was carefully extracted for sectioning and hematoxylin-eosin (H&E) staining. The processes of brain tissue sectioning, H&E staining, and slide scanning were conducted by Biossci Company (Wuhan, China).

### 2.10. Establishment and Monitoring of Orthotopic Glioma Models

On day 0, glioma cells stably transfected with the luciferase gene were digested, washed and then resuspended at a density of 50,000 cells per mouse. The cell suspension was intracranially injected into the right hemisphere of anesthetized mice using a stereotactic instrument (68801, RWD, Shenzhen, China). On days 10, 14, and 18, each mouse received 200,000 dendritic cell vaccines via tail vein injection. On day 14, anesthetized mice were intraperitoneally injected with 150 mg/kg D-luciferin sodium (HY-12591, MCE, Shanghai, China). Bioluminescence intensity in the brain was monitored using a small animal in vivo imaging system (LAGO X, Spectral Instruments Imaging, Tucson, AZ, USA) within 10–40 min post-injection. Mice surviving beyond day 45 without significant symptoms underwent tumor reimplantation. Bioluminescence signals were reassessed on day 59. The longest diameter (L) and the perpendicular shortest diameter (W) of the tumor region were measured in the scanned sections using CaseViewer software (version 2.4.0.119028), and the tumor volume was estimated using the formula V = 1/2 × L × W^2^.

### 2.11. Protein Sample Preparation

Dendritic cells were pelleted by centrifugation, and the supernatant was carefully aspirated and discarded. The cell pellet was then resuspended in phosphate-buffered saline (PBS) and washed twice to thoroughly remove any residual culture medium. A working solution of RIPA lysis buffer was prepared by supplementing it with phenylmethylsulfonyl fluoride at a recommended ratio (typically 100:1, *v*/*v*) to inhibit protease activity. This mixture was added to the cell pellet, followed by incubation on ice for 30 min with intermittent vortexing to ensure complete lysis. The lysate was subsequently centrifuged at 12,000× *g* for 15 min at 4 °C to precipitate insoluble debris. The resulting supernatant, containing the soluble protein fraction, was carefully collected. The total protein concentration was quantified using a bicinchoninic acid (BCA) protein assay kit (G2026, ServiceBio, Wuhan, China), according to the manufacturer’s instructions. Finally, the protein samples were mixed with protein loading buffer (WB2001, NCM Biotech, Suzhou, China) at an appropriate volume ratio (typically 1:4, sample/buffer) and denatured by boiling at 95–100 °C for 5–10 min to ensure complete dissociation and linearization of the proteins for subsequent electrophoretic analysis.

### 2.12. Western Blot Analysis

The prepared protein samples were loaded onto an SDS-PAGE gel for electrophoretic separation. Following electrophoresis, the proteins were transferred onto a methanol-activated PVDF membrane (IPFL00005, Millipore, Burlington, MA, USA) using a wet transfer system. After successful transfer, the membrane was blocked with 5% bovine serum albumin to prevent non-specific binding. The blocked membrane was then washed and incubated overnight at 4 °C with the following primary antibodies: TLR3 (A11778, ABclonal, Wuhan, China), p38 MAPK (14064-1-AP, Proteintech, Wuhan, China), phospho-p38 MAPK (28796-1-AP, Proteintech, Wuhan, China), ATF3 (A13469, ABclonal, Wuhan, China), GAPDH (10494-1-AP, Proteintech, Wuhan, China), and β-actin. After primary antibody incubation, the membrane was thoroughly washed and subsequently incubated with species-appropriate HRP-conjugated secondary antibodies. Following a final series of washes, the membrane was treated with a chemiluminescent substrate (P10060, NCM Biotech, Suzhou, China) according to the manufacturer’s instructions. The immunoreactive signals were detected and visualized using the GeneGnome GRQ imaging system (Syngene, Cambridge, UK).

### 2.13. Enzyme-Linked Immunosorbent Assay (ELISA)

Supernatants were collected from dendritic cell cultures following centrifugation. According to the manufacturer’s instructions provided with the Mouse IL-12 (p70) ELISA Kit (EK0422, Boster, Wuhan, China) or Human IL-12 (p70) ELISA Kit (EK0421, Boster, Wuhan, China), the supernatants, a series of diluted standard solutions, and sample dilution buffer were aliquoted into the pre-coated wells of a microplate. The plate was sealed with an adhesive membrane and incubated for 90 min. After incubation, the liquid was aspirated, and a biotin-conjugated detection antibody working solution was added to each well, followed by a 60-min incubation. The wells were then thoroughly washed, and an Avidin-Biotin-Peroxidase Complex (ABC) working solution was added, with subsequent incubation for 30 min. Following another washing step, Tetramethylbenzidine (TMB) substrate solution was added, and the plate was incubated for 20 min in the dark to allow color development. The enzymatic reaction was terminated by adding stop solution. The optical density (O.D.) of each well was immediately measured at a wavelength of 450 nm using a microplate reader. A standard curve was generated by plotting the mean O.D. values of the standard concentrations versus their corresponding concentrations. The concentration of IL-12 in each test sample was determined by interpolating its O.D. value from the standard curve.

### 2.14. Flow Cytometry

Cells were harvested and washed thoroughly with phosphate-buffered saline (PBS). According to the manufacturer’s instructions (423106, BioLegend, San Diego, CA, USA), an appropriate amount of flow cytometry antibody was added to the cell suspension. The mixture was thoroughly mixed and incubated protected from light. After incubation, cells were washed again with PBS to remove unbound antibodies. For samples requiring intracellular staining, after the aforementioned steps, cells were fixed using FluoroFix™ Buffer (422101, BioLegend, San Diego, CA, USA). Fixed cells were washed with diluted 10× Intracellular Staining Permeabilization Wash Buffer (421002, BioLegend, San Diego, CA, USA). Subsequently, the corresponding intracellular flow antibody was added, and the mixture was thoroughly mixed and incubated for 60 min protected from light. After incubation, cells were washed again with Intracellular Staining Permeabilization Wash Buffer and resuspended. The prepared samples were analyzed on a CytoFLEX flow cytometer (Beckman Coulter, Indianapolis, IN, USA). The acquired data were statistically processed and analyzed using FlowJo software (version 10.9.0).

### 2.15. Transcriptome Sequencing and Bioinformatic Analysis

Mature dendritic cells (DCs) cultured in vitro were distributed into six independent culture dishes. Three dishes were designated as the untreated control group, while the other three were stimulated with a combination of 10 ug/mL CpG ODN and 10 ug/mL poly(I:C) for 24 h. Total RNA was subsequently extracted from all cell samples for transcriptome sequencing. The sequencing was performed on an Illumina platform, with an average sequencing data volume of approximately 6 Gb per sample to ensure sufficient transcriptome coverage. The Q30 base calling accuracy rate for all samples exceeded 85%, guaranteeing the reliability of the raw data. Bioinformatic analysis followed a standardized pipeline. The raw sequencing data were first subjected to quality control filtering using fastp software (version 0.23.4) to remove adapter sequences and low-quality reads. The clean data were then aligned to the appropriate reference genome using the HISAT2 software (version 2.2.1). Gene expression levels were quantified using HTSeq software (version 0.11.2), presented as raw read counts, and further converted into FPKM values for cross-sample comparisons. Differential expression analysis was conducted using the DESeq2 R package (version 1.36.0). Its internal pipeline includes a normalization step that corrects for sequencing depth variation in the raw read counts. Significantly differentially expressed genes were identified using a threshold of an adjusted *p*-value less than 0.05 and an absolute log2 fold change greater than 1. Based on this analysis, a volcano plot was constructed to visualize the overall distribution of differential genes. For genes significantly upregulated in the combination treatment group, KEGG pathway enrichment analysis was performed using the cluster Profiler tool. The enriched pathways were ranked according to the adjusted *p*-value of the enrichment results. The top 20 most significantly enriched signaling pathways were selected, and their enrichment significance along with the expression patterns of associated genes were visualized using a heatmap.

### 2.16. Statistical Analysis

All data analyses and visualizations were performed using GraphPad Prism software (version 9.0.0). Data are presented as the mean ± standard deviation, and individual data points are displayed on column bar graphs. Spearman’s correlation analysis was employed to assess the relationship between two variables. For comparisons between multiple groups, one-way analysis of variance (ANOVA) was conducted, while an unpaired Student’s *t*-test was used for comparisons between two groups. Statistical significance was defined as a *p*-value less than 0.05. Specific significance levels were denoted as follows: * = *p* < 0.05, ** = *p* < 0.01, *** = *p* < 0.001, and **** = *p* < 0.0001.

## 3. Results

### 3.1. Synergistic Activation of TLR3 and TLR9 Alters Dendritic Cell Activity In Vitro

**Figure 1:**
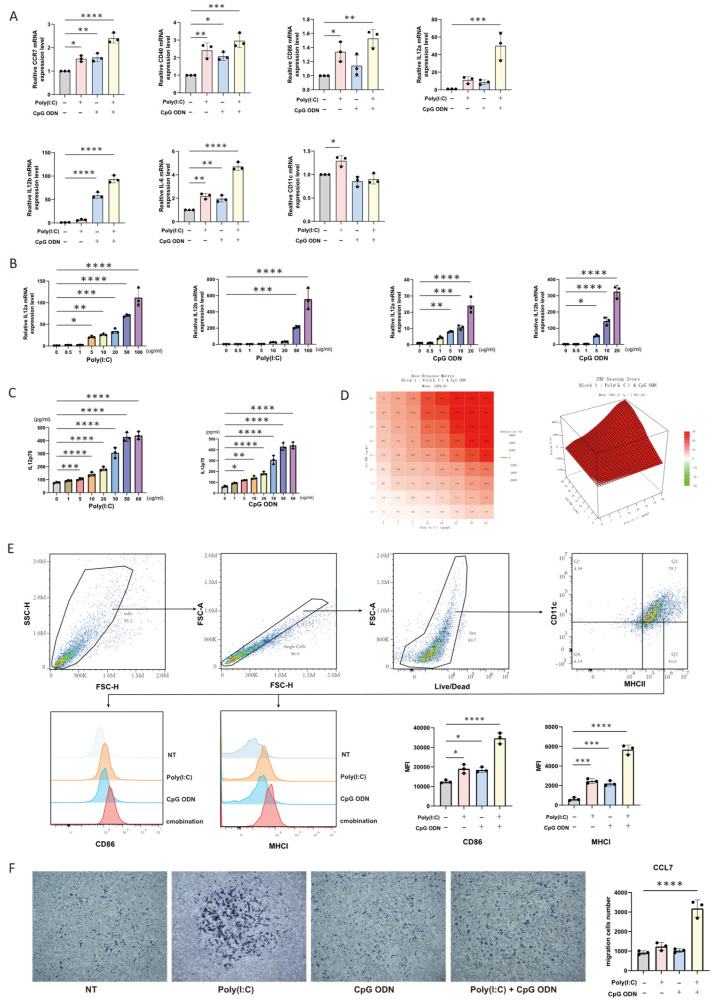
Combined Stimulation with Poly(I:C) and CpG ODN Alters the Activity of Mouse Bone Marrow-Derived Dendritic Cells (BMDCs). (**A**) Changes in the mRNA expression levels of genes associated with the biological functions of BMDCs were measured using quantitative real-time PCR (qRT-PCR). Cells were treated with 5 ug/mL poly(I:C), 5 ug/mL CpG ODN, or their combination for 12 h. (**B**) Relative gene expression levels of IL-12a and IL-12b in BMDCs stimulated with individually titrated concentrations of poly(I:C) or CpG ODN were assessed by qRT-PCR. (**C**) IL-12p70 protein secretion levels were quantified by enzyme-linked immunosorbent assay (ELISA) in BMDCs treated with individually titrated concentrations of poly(I:C) or CpG ODN. The left and right panels of the figure show the levels of IL-12p70 measured in the supernatant after 24 h of treatment with different concentrations of poly(I:C) and CpG ODN, respectively. (**D**) SynergyFinder analysis was used to calculate the synergy scores for the upregulation of IL-12p70 protein secretion following combined stimulation with CpG ODN and poly(I:C) across a matrix of concentrations. A synergy score >100 indicates a synergistic effect, while a score < −100 suggests antagonism. Higher scores or increasingly intense colors represent stronger effects. Two-dimensional (left) and three-dimensional (right) visualizations of the interaction data are shown. (**E**) Flow cytometric analysis of the MFI of CD86 and MHC-I in purified BMDCs under different treatment conditions (untreated, 10 ug/mL poly(I:C), 10 ug/mL CpG ODN, or their combination). Overlay histograms (left) and quantitative bar graphs (right) of the MFI data are presented. (**F**) Transwell migration assay was performed to evaluate the chemotactic capacity of treated BMDCs toward the lower chamber pre-coated with 2 ug/mL CCL7. Representative micrographs (left) captured under a 10× microscope and quantitative analysis (right) of cell migration are shown. **** *p* < 0.0001; *** *p* < 0.001; ** *p* < 0.01; * *p* < 0.05.

Previous studies have reported that poly(I:C) and CpG ODN can inhibit the tumor-promoting effects of microglia in the central nervous system. Since both dendritic cells (DCs) and macrophages are professional antigen-presenting cells, we further investigated the mRNA expression levels of key immunomodulatory genes following 12 h of stimulation with 5 ug/mL CpG ODN and 10 ug/mL poly(I:C), either individually or in combination (Figure 1A). The genes analyzed included CD11c (associated with DC maturation), Cd40 and Cd86 (involved in T-cell co-stimulation), Ccr7 (related to DC migration), Il12a/Il12b (critical for T-cell differentiation and maturation), and IL-6 (a pro-inflammatory cytokine). Compared to the control group, all genes except CD11c and Il12a were upregulated upon individual treatment with poly(I:C) or CpG ODN. In the combination treatment group, all genes except CD11c showed significant upregulation. Given the critical role of IL-12 in DC-mediated T-cell maturation and functional activation, we extended our analysis to include multiple concentration gradients of individual agonists to assess IL12-a and IL12-b expression levels after 12 h of stimulation (Figure 1B). A dose-dependent increase in gene expression was observed with both agonists. To determine whether these transcriptional changes translated to enhanced protein secretion, we measured IL-12 production by ELISA after 36 h of stimulation with individual (Figure 1C) or combined agonists across a range of concentrations. The resulting matrix data were analyzed using the ZIP algorithm in SynergyFinder to quantify synergistic effects (Figure 1D). The heatmap (left) and 3D surface plot (right) illustrate synergy scores, where a score > 100 indicates synergy and a score < −100 indicates antagonism. To validate the significance of the synergistic effect, we performed a two-way ANOVA on the synergy scores calculated from three independent datasets, which yielded an interaction *p*-value of < 0.0001. The corresponding two-way ANOVA statistical table generated by GraphPad Prism has been uploaded in Excel format. Since CD86 and MHC-I are essential for DC-mediated cross-presentation and T-cell activation, we evaluated their expression levels by flow cytometry after 36 h of stimulation with 10 ug/mL poly(I:C) and/or 10 ug/mL CpG ODN (Figure 1E). Both individual treatments increased the mean fluorescence intensity (MFI) of CD86 and MHC-I compared to the control, and the combination treatment resulted in a further significant enhancement. DC migratory capacity is a key indicator of antitumor efficacy. Using a Transwell assay, we evaluated the migration of DCs under four conditions: untreated, pre-treated with 10 ug/mL poly(I:C) for 24 h, pre-treated with 10 ug/mL CpG ODN for 24 h, or pre-treated with both agonists. The lower chamber contained 2 ug/mL CCL7 as a chemoattractant (Figure 1F). Compared to the control group, treatment with individual agonists failed to enhance the migratory capacity of dendritic cells, while the combined treatment led to an increase in migratory ability.

### 3.2. Synergistically Activated Dendritic Cells Enhance the Biological Functions of CD8^+^ T Cells

**Figure 2:**
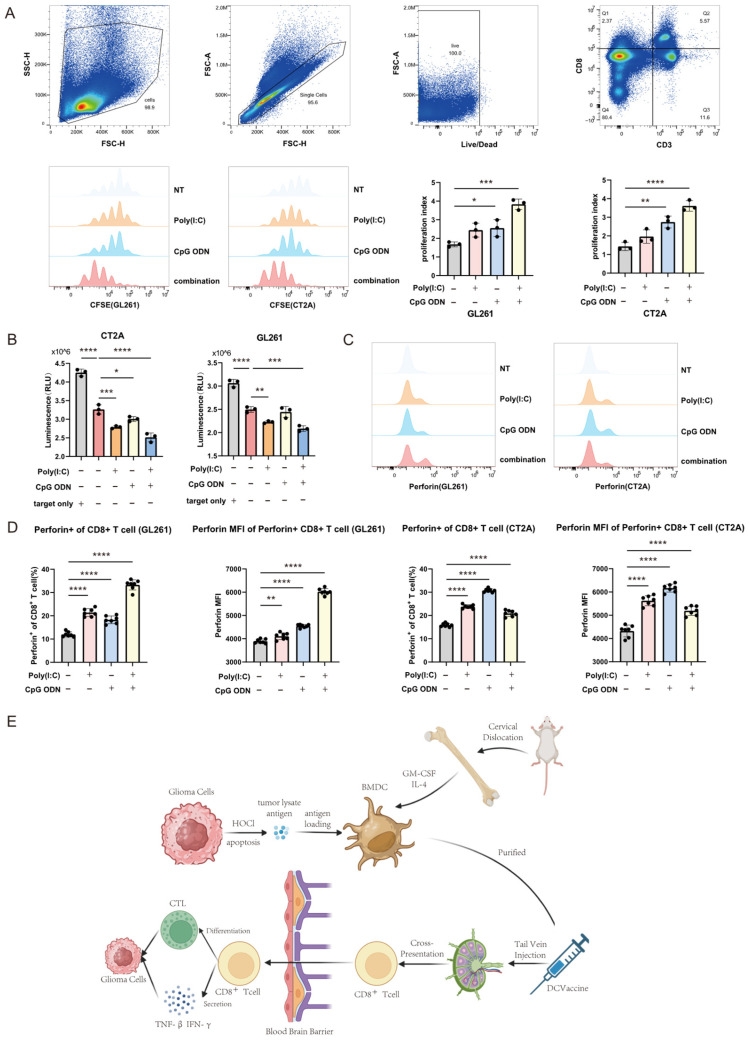
Synergistically Activated Dendritic Cells Enhance the Biological Functions of CD8^+^ T Cells. (**A**) Proliferation of CFSE-labeled mouse splenic CD8^+^ T cells following co-culture with dendritic cells (DCs) was assessed by flow cytometry. Overlay histograms (left) and quantitative analysis of the proliferation index (right) are shown. (**B**) Cytolytic activity of activated CD8^+^ T cells was evaluated by adding luciferase gene-transfected glioma cells into the co-culture system described in (**A**). After 24 h, cells were lysed, and bioluminescence intensity was measured to quantify tumor cell killing. (**C**) Expression levels of surface activation markers on mouse splenic CD8^+^ T cells after co-culture with DCs. Representative overlay histograms from flow cytometric analysis are displayed. (**D**) Quantitative analysis of the mean fluorescence intensity (MFI) of surface markers shown in panel (**C**). (**E**) Schematic illustration of the preparation and administration of bone marrow-derived dendritic cell (BMDC) vaccines in mice. **** *p* < 0.0001; *** *p* < 0.001; ** *p* < 0.01; * *p* < 0.05.

Since dendritic cell (DC) vaccines exert their antitumor effects by presenting loaded antigens to and activating CD8^+^ T cells in vivo, we designed a series of in vitro experiments to evaluate the T cell activating capacity of DCs following pharmacological pretreatment. Four groups of DCs were established: untreated control, pretreated with 10 ug/mL poly(I:C) for 24 h, pretreated with 10 ug/mL CpG ODN for 24 h, and pretreated with both 10 ug/mL poly(I:C) and 10 ug/mL CpG ODN for 24 h. These DCs were loaded with GL261 or CT2A glioma cell lysates and then co-cultured with CFSE-labeled mouse splenic lymphocytes. T cell proliferation was assessed by flow cytometry (Figure 2A, top: gating strategy; bottom left: overlay histograms of CD8^+^ T cell proliferation; bottom right: quantitative proliferation index). While DCs pretreated with poly(I:C) did not significantly enhance CD8^+^ T cell proliferation compared to the control group in experiments using either cell line, DCs pretreated with CpG ODN alone markedly promoted CD8^+^ T cell proliferation. This effect was further significantly enhanced in the combination treatment group. To evaluate the cytolytic function of activated CD8^+^ T cells, a killing assay was performed. Luciferase-transfected glioma cells were added to the co-culture system described in Figure 2A. After a specified incubation period, cells were lysed, and bioluminescence intensity was measured to quantify tumor cell killing (Figure 2B, left: CT2A; right: GL261). Activated CD8^+^ T cells mediate tumor cell apoptosis through the release of cytotoxic molecules such as perforin and via Fas signaling pathways. We used flow cytometry to measure the MFI of perforin and the percentage of perforin-positive cells among CD8^+^ T cells across experimental groups (Figure 2C: overlay histograms; Figure 2D: quantitative analysis of the data from Figure 2C). Compared to the control group, both the percentage of perforin-positive CD8^+^ T cells and the perforin MFI were increased in groups stimulated with poly(I:C) or CpG ODN alone. These metrics were further significantly elevated in the combination treatment group, indicating a synergistic enhancement of CD8^+^ T cell cytotoxic potential. Figure 2E presents a schematic diagram illustrating the preparation and mechanism of action of the dendritic cell vaccine for glioma in this study.

### 3.3. Synergistically Activated Dendritic Cell Vaccines Reduce Tumor Volume and Delay Glioma Progression in Mice

**Figure 3:**
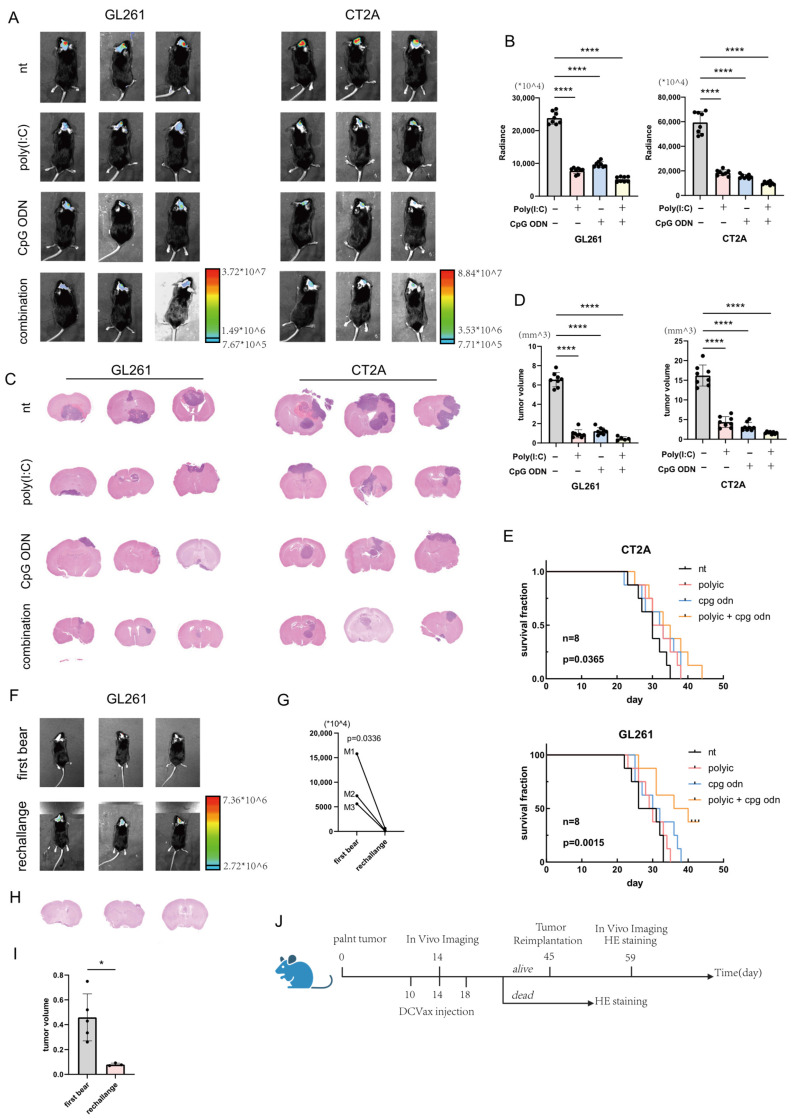
Activated Dendritic Cell Vaccines Improve Survival in Glioma-Bearing Mice. (**A**) In vivo bioluminescence imaging of orthotopic glioma models. Tumor bioluminescence intensity was measured on day 14 post-implantation of luciferase gene-transfected glioma cell lines in 6–10-week-old male C57BL/6 mice. (**B**) Quantitative analysis of the bioluminescence data presented in panel (n = 8) (**A**). (**C**) Representative scanned images of hematoxylin and eosin (H&E)-stained brain tissue sections from tumor-bearing mice. Tissues were collected upon the onset of tumor-related symptoms or a moribund state. (**D**) Quantitative analysis of tumor volumes calculated from H&E-stained sections using the formula V = 1/2 × L × W^2^ (GL261 combination treatment group n = 5, other groups n = 8). (**E**) Kaplan–Meier survival curve of glioma-bearing mice. (**F**) In vivo bioluminescence images of tumor recurrence in rechallenged mice. Three mice bearing GL261 tumors survived beyond day 45 without clinical symptoms and were rechallenged with tumor cells. Upper panel: bioluminescence images taken 14 days after the initial implantation; Lower panel: bioluminescence images of the same three mice 14 days after the second implantation. (**G**) Quantitative analysis of the bioluminescence data shown in panel (**F**). (**H**) Representative scanned images of H&E-stained brain sections from the three rechallenged mice in panel (**F**). (**I**) Comparative analysis of tumor volumes from H&E-stained brain sections between the three rechallenged mice (second implantation) and five mice that underwent only a single tumor implantation (first bear group n = 5, rechallenge group n = 3). (**J**) Timeline of experimental procedures for in vivo evaluation. Schematic diagram illustrating the key time points for tumor implantation, intravenous administration of the vaccine, secondary tumor challenge, and collection of brain tissue in the orthotopic glioma mouse model. **** *p* < 0.0001; * *p* < 0.05.

To evaluate the in vivo efficacy of dendritic cell (DC) vaccines modified with combined agonist treatment, orthotopic glioma models were established in 8-week-old male C57BL/6 mice. On day 0, mice were intracranially implanted with luciferase gene-transfected glioma cell lines (GL261 or CT2A). On days 10, 14, and 18, DC vaccines loaded with whole tumor lysate antigens were administered via tail vein injection. These DCs were pretreated under one of four conditions prior to antigen loading: no treatment, 10 ug/mL poly(I:C) for 24 h, 10 ug/mL CpG ODN for 24 h, or a combination of both poly(I:C) and CpG ODN (each at 10 ug/mL for 24 h) (Figure 3A shows representative in vivo bioluminescence images; Figure 3B provides quantitative analysis of the bioluminescence data derived from Figure 3A, n = 8). Compared to the control group, both individual treatment groups and the combination group exhibited a significant reduction in bioluminescence intensity in the head region, indicating suppressed tumor growth. Brain tissues were harvested from tumor-bearing mice upon the onset of clinical symptoms or imminent mortality for histological analysis. Sections were stained with hematoxylin and eosin (H&E), and tumor volume was calculated using the formula V = 0.5 × L× W^2^, followed by quantitative visualization (Figure 3C,D, GL261 combination treatment group n = 5, other groups n = 8). Both monotherapy and combination therapy groups showed a significant reduction in tumor volume compared to the control. Furthermore, survival curves were plotted for mice bearing GL261 or CT2A tumors (Figure 3E, n = 8). The statistics passed Logrank test for trend, CT2A: *p* = 0.0365 GL261: *p* = 0.0015. The combination treatment group exhibited a prolonged survival time compared to both the monotherapy and control groups. Notably, in the GL261-bearing group, three mice from the combination treatment group survived beyond 45 days without apparent clinical symptoms. These mice were rechallenged with tumor cells, and in vivo imaging was performed 14 days later to assess recurrent tumor size (Figure 3F: representative images; Figure 3G: quantitative analysis). The bioluminescence signal after the second implantation was significantly weaker compared to the initial implantation. Following imaging, brain tissues from these three rechallenged mice were sectioned and subjected to H&E staining. Tumor sizes were calculated and analyzed quantitatively (Figure 3H,I, first bear group n = 5, rechallenge group n = 3). Compared to the five mice from the same treatment group that succumbed during the initial implantation (Figure 3A), the three rechallenged mice displayed significantly smaller tumor volumes. These findings suggest that DC vaccines modified with combined TLR agonist treatment may play a positive role in preventing glioma recurrence. Figure 3J is a schematic diagram illustrating the treatment protocol and the respective time points for different procedures in the glioma mouse model.

### 3.4. TLR9-Dependent p38 MAPK-ATF3 Signaling Promotes TLR3 Upregulation and Mediates Synergistic Activation

**Figure 4:**
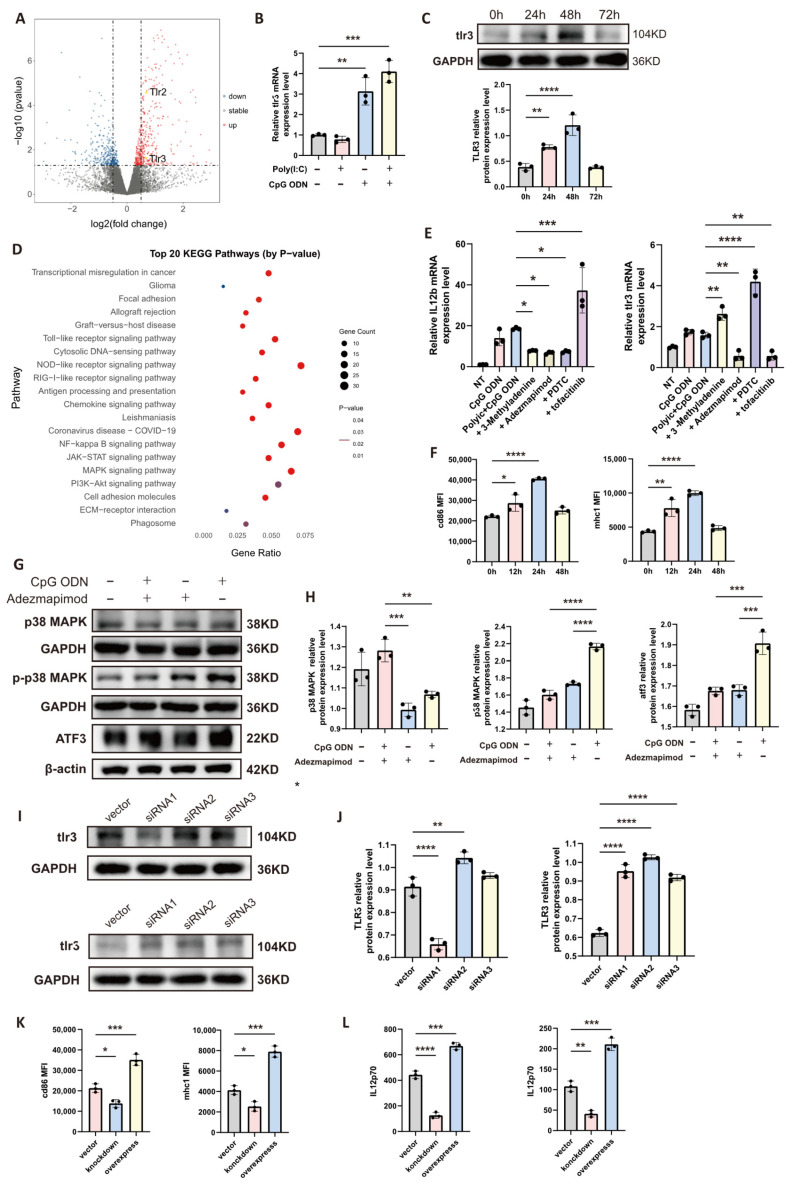
CpG ODN Activates the p38 MAPK-ATF3 Pathway and Upregulates TLR3 Expression. (**A**) Volcano plot derived from high-throughput transcriptome sequencing analysis, comparing gene expression profiles between the combination treatment group and the control group. TLR2 and TLR3 are highlighted among the significantly upregulated genes. (**B**) Relative tlr3 gene expression levels in dendritic cells across four experimental groups were quantified using quantitative real-time PCR (qRT-PCR). (**C**) Western blot analysis of TLR3 protein expression in dendritic cells following stimulation with CpG ODN over a time course. (**D**) Top 20 significantly enriched KEGG pathways identified through enrichment analysis of the transcriptome data. Sphere color indicates the statistical significance (*p*-value) of the corresponding pathway. (**E**) Relative mRNA expression levels of IL12B and TLR3 were measured by qRT-PCR in dendritic cells treated under various conditions: control, CpG ODN alone, combination treatment (CpG ODN + poly(I:C)), and combination treatment supplemented with specific pathway inhibitors—Tofacitinib (JAK inhibitor), PDTC (NF-kB inhibitor), 3-methyladenine (PI3K inhibitor), and Adezmapimod (p38 MAPK inhibitor). (**F**) Bone marrow-derived dendritic cells were pretreated with 10 μg/mL CpG ODN for 0, 12, 24, and 48 h. Subsequently, poly(I:C) was added to the culture, which was then continued for an additional 24 h prior to analysis by flow cytometry. Statistical results for the mean fluorescence intensity of CD86 and MHC-I in dendritic cells are shown in the figure. (**G**) Western blot analysis validating the suppressive effect of Adezmapimod on the p38 MAPK-ATF3 signaling pathway. (**H**) Quantitative analysis of the data presented in panel (**G**). (**I**) Western blot verification of successful TLR3 knockdown or overexpression in dendritic cells using a transient transfection system. Top: knockdown efficiency; Bottom: overexpression efficiency. (**J**) Quantitative analysis of the data from panel (**I**). Left: knockdown groups; Right: overexpression groups. (**K**) Flow cytometric analysis of CD86 and MHC-I expression levels in dendritic cells subjected to TLR3 knockdown or overexpression, followed by activation with 10 ug/mL CpG ODN and 10 ug/mL poly(I:C) for 36 h. (**L**) Secretion levels of IL-12 and IFN-γ were measured by ELISA in dendritic cells subjected to TLR3 knockdown or overexpression, followed by activation with 10 ug/mL CpG ODN and 10 ug/mL poly(I:C) for 48 h. **** *p* < 0.0001; *** *p* < 0.001; ** *p* < 0.01; * *p* < 0.05.

Previous experimental findings suggested that the enhanced antitumor efficacy observed upon combined activation of dendritic cells (DCs) with poly(I:C) and CpG ODN might be mediated by underlying synergistic signaling mechanisms. To investigate this, we performed transcriptome sequencing on DCs subjected to two conditions: untreated control and combined stimulation with 10 ug/mL poly(I:C) and 10 ug/mL CpG ODN for 12 h. Volcano plot analysis of the sequencing data revealed significant upregulation of TLR3 and ATF3 expression in the combination treatment group compared to the untreated control (Figure 4A). Given that ATF3 is involved in Toll-like receptor 9 (TLR9) signal transduction, we hypothesized that TLR9 activation might lead to the upregulation of TLR3 expression, thereby mediating the observed synergy. To validate this, we measured the relative expression levels of TLR3 in four experimental groups: untreated control, 10 ug/mL poly(I:C) alone, 10 ug/mL CpG ODN alone, and the combination treatment (10 ug/mL poly(I:C) + 10 ug/mL CpG ODN) after 12 h of stimulation (Figure 4B). Both the CpG ODN alone and combination treatment groups exhibited increased TLR3 expression. At the protein level, Western blot analysis was employed to assess TLR3 protein expression in DCs pre-activated with CpG ODN over a time course of 0 h (untreated), 24 h, 48 h, and 72 h (Figure 4C). TLR3 protein levels were significantly elevated at 24 h and 48 h compared to baseline (0 h), but returned to levels not significantly different from baseline by 72 h. To elucidate the downstream pathways activated by TLR9, we performed KEGG pathway enrichment analysis on the transcriptome data and ranked the top 20 significantly enriched pathways based on *p*-value (Figure 4D). Several pathways critically involved in Toll-like receptor signaling—including JAK-STAT, NF-kB, PI3K-Akt, and MAPK were among the top 20 enriched. We therefore selected specific inhibitors for these pathways: Tofacitinib (JAK inhibitor), PDTC (NF-kB inhibitor), 3-methyladenine (3-MA, PI3K inhibitor), and Adezmapimod (p38 MAPK inhibitor). Using IL12B expression as a key readout for antitumor efficacy, we designed seven experimental groups, as schematically represented in Figure 4E, to treat DCs with agonists and/or inhibitors for 12 h before measuring the relative expression levels of IL12B and TLR3. Compared to the combination treatment group, the groups additionally treated with Adezmapimod (p38 MAPK pathway inhibitor) showed reduced expression of both IL-12b and TLR3, suggesting that p38 MAPK signaling pathways are upstream regulators contributing to the upregulation of TLR3 and IL-12b. To preliminarily investigate whether upregulation of the TLR3 receptor could affect the protein levels of antitumor-related molecules in dendritic cells, we performed flow cytometry analysis on sequentially treated cells (Figure 4F). To further verify that TLR9 activation promotes TLR3 upregulation through the p38 MAPK -ATF3 axis, we designed a Western blot experiment (Figure 4G) and quantified the results (Figure 4H). When measuring total p38 MAPK and phosphorylated p38 MAPK (p-p38) with GAPDH as a loading control, the group treated with CpG ODN + Adezmapimod showed a slight increase in total p38 MAPK but a decrease in p-p38 levels compared to the group treated with CpG ODN alone. Furthermore, ATF3 expression (normalized to β-actin) was reduced in the CpG ODN + Adezmapimod group compared to the CpG ODN-only group. These results indicate that CpG ODN activates p38 phosphorylation and ATF3 expression, and that Adezmapimod inhibits this activation. Finally, to functionally characterize this pathway, we used a liposome-based transient transfection system to knock down or overexpress ATF3 in bone marrow-derived DCs (BMDCs) (Figure 4I, top: knockdown; bottom: overexpression; Figure 4J provides quantitative analysis). Vector control, knockdown, and overexpression groups were then treated with the combination of agonists, and assessed by flow cytometry for the mean fluorescence intensity (MFI) of CD86 and MHC class I (Figure 4K), and by ELISA for the secretion levels of IL-12 and IFN-γ (Figure 4L). The knockdown group showed slightly lower MFI for CD86 and MHC I compared to the vector control group, while the overexpression group exhibited higher MFI levels. Similarly, secretion of IL-12 and IFN-γ was lower in the knockdown group and higher in the overexpression group compared to the vector control. Collectively, these data demonstrate that ATF3 expression levels directly influence the activation state and effector functions of DCs, and that the TLR9-p38 MAPK-ATF3 signaling axis plays a pivotal role in the synergistic upregulation of TLR3 and enhancement of antitumor immunity.

### 3.5. Sequential Agonist Treatment Enhances the Antitumor Efficacy of Dendritic Cell Vaccines

**Figure 5:**
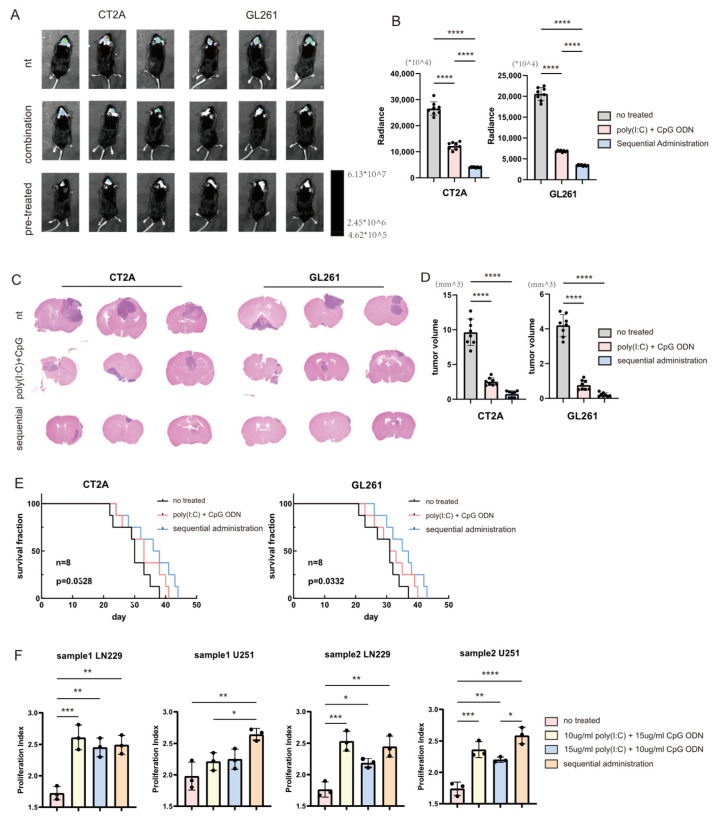
Sequential Administration of Activated Dendritic Cell Vaccines Enhances Antitumor Efficacy in Orthotopic Glioma Models. (**A**) In vivo bioluminescence imaging of tumor-bearing mice. DCs were pre-treated under one of three conditions: untreated control, simultaneous combination of agonists (combination treatment), or sequential agonist stimulation (sequential treatment), followed by loading with glioma cell line-derived antigens. (**B**) Quantitative analysis of the bioluminescence intensity data presented in panel (**A**) (n = 8). (**C**) Representative scanned images of hematoxylin and eosin (H&E)-stained brain sections from mice harvested upon the onset of clinical symptoms or a moribund state. (**D**) Quantitative analysis of the tumor volume data presented in panel (**C**) (n = 8). (**E**) Kaplan–Meier survival curve of glioma-bearing mice across different treatment groups (n = 8). (**F**) Proliferation of CD8^+^ T cells following in vitro co-culture with human-derived engineered dendritic cell (DC) vaccines. **** *p* < 0.0001; *** *p* < 0.001; ** *p* < 0.01; * *p* < 0.05.

Based on the finding that TLR3 upregulation following TLR9 activation requires a specific time interval, we hypothesized that a sequential administration protocol with an optimal interval would yield superior therapeutic outcomes compared to simultaneous combination treatment. To test this, orthotopic glioma models were established in mice by intracranial implantation of luciferase gene-transfected GL261 or CT2A glioma cell lines on day 0. On days 10, 14, and 18, dendritic cell (DC) vaccines loaded with whole tumor lysate antigens derived from the respective glioma cell lines were administered via tail vein injection. These DCs were subjected to one of three pretreatment conditions before antigen loading: no treatment (control), simultaneous stimulation with 10 ug/mL poly(I:C) + 10 ug/mL CpG ODN for 24 h, or sequential stimulation involving pretreatment with 10 ug/mL CpG ODN for 24 h followed by 10 ug/mL poly(I:C) for another 24 h. In vivo bioluminescence imaging (Figure 5A) and subsequent quantitative analysis (Figure 5B) revealed that mice receiving DCs pretreated with the sequential regimen (pre-treated group) exhibited significantly lower bioluminescence signals in the brain region compared to those receiving DCs from the simultaneous combination group (n = 8). Upon the onset of clinical symptoms or a moribund state, brain tissues were harvested for histological examination. Sections were stained with hematoxylin and eosin (H&E), scanned, and tumor volumes were calculated and quantitatively analyzed (Figure 5C: representative images; Figure 5D: quantitative data, n = 8). The sequential treatment group demonstrated a significant reduction in tumor volume compared to the simultaneous treatment group. Furthermore, survival was monitored, and Kaplan–Meier curves were plotted (Figure 5E, n = 8). The statistics passed Logrank test for trend, CT2A: *p* = 0.0328 GL261: *p* = 0.0332. Mice in the sequential treatment group exhibited a significantly prolonged overall survival compared to those in the simultaneous treatment group. Collectively, these results demonstrate that a sequential TLR agonist activation strategy, which accommodates the temporal dynamics of TLR3 upregulation, significantly enhances the antitumor efficacy of DC vaccines, leading to superior tumor control and improved survival outcomes in murine glioma models. The aforementioned experiments confirmed that the sequential treatment regimen elicited superior antitumor effects in murine models. To evaluate the translational potential of this approach for clinical application, we conducted an in vitro co-culture system using human-derived engineered dendritic cell (DC) vaccines and autologous CD8^+^ T cells purified from the same healthy volunteers. The engineered DCs were pre-labeled with CFSE dye, and the proliferation of CD8^+^ T cells was assessed after a specified co-culture period. As illustrated in Figure 5F, engineered DCs and peripheral blood T cells from two volunteers were tested against the LN229 and U251 glioma cell lines. The experimental design included four groups: Engineered DCs without any treatment (untreated control); Engineered DCs treated simultaneously with 10 ug/mL poly(I:C) + 15 ug/mL CpG ODN for 24 h; Engineered DCs treated simultaneously with 15 ug/mL poly(I:C) + 10 ug/mL CpG ODN for 24 h; Engineered DCs subjected to sequential treatment: pretreatment with 10 ug/mL CpG ODN for 24 h followed by 10 ug/mL poly(I:C) for another 24 h. The results demonstrated that the sequential treatment group exhibited a significantly enhanced capacity to stimulate CD8^+^ T cell proliferation compared to the other groups. Notably, this superior effect was achieved with a lower total agonist dosage, indicating that the sequential strategy provides a more potent immunostimulatory outcome than simultaneous combination treatment.

## 4. Discussion

In the investigation of immunotherapeutic strategies for glioma, our in vitro experiments demonstrated that individual application of the Toll-like receptor 9 (TLR9) agonist CpG ODN or the TLR3 agonist poly(I:C) moderately upregulates the expression of certain antitumor-related genes (e.g., CD86, MHC-I, IL-12) in dendritic cells (DCs). However, the magnitude of this upregulation was relatively limited. Notably, a synergistic enhancement in the expression of these immunologically active markers was observed when a combined agonist strategy was employed, with the effect being substantially superior to that of any single agonist treatment. This synergistic effect is likely attributable to cross-talk between TLR signaling pathways. Of particular significance, our mechanistic exploration suggests that TLR9 activation may indirectly upregulate TLR3 expression via the p38 MAPK–ATF3 signaling axis, thereby amplifying the responsiveness of DCs to subsequent TLR3 stimulation. This likely constitutes a crucial molecular basis for the synergy observed with combined treatment. ATF3 (activating transcription factor 3) has conventionally been characterized in oncological research as a stress-inducible transcription factor that typically exerts a negative regulatory influence on the p38 MAPK pathway within cells, potentially suppressing tumor growth and invasion [21]. Similarly, our study reveals a divergent role for ATF3 in DCs: it functions as a key intermediary downstream of TLR9, facilitating the upregulation of TLR3. Leveraging this property, we implemented a sequential activation strategy (CpG ODN followed by poly(I:C)), which significantly augmented the adjuvant effect of DC vaccines, thereby potentiating the antitumor immune response. In in vivo experiments, mice administered DC vaccines pre-activated synergistically exhibited significant tumor growth suppression and prolonged survival. The mechanism underlying this efficacy involves the potent activation of peripheral lymphocytes by these activated DC vaccines, promoting their proliferation and differentiation, which consequently improves the prognosis of mice bearing central nervous system (CNS) gliomas. This finding aligns with a research confirming the presence of resident T cells within the CNS and their role in immune surveillance [22]. Nevertheless, the observed infiltration of T cells into the CNS in an orthotopic glioma model could also be partially ascribed to the compromise of the blood–brain barrier (BBB) resulting from tumor growth [23,24]. Additionally, our histological analysis indicated that tumors with significant hemorrhagic foci demonstrated relatively constrained growth and a delayed onset of clinical symptoms. This suggests that alterations in the vascular microenvironment (e.g., inflammatory reactions or antigen release potentially triggered by hemorrhage) may influence the potency or nature of the antitumor immune response. In the final phase of our research, we employed an engineered dendritic cell (DC) line. Its paramount advantage lies in its capacity for unlimited proliferation in vitro (immortalization), and even in the absence of TLR3 or TLR9 agonists, its baseline expression of immunologically active markers (e.g., CD80, CD86, MHC-II) was significantly higher than that of DCs conventionally differentiated from peripheral blood mononuclear cells (PBMCs). Both the sequential TLR9 and TLR3 activation protocol and such engineered DC vaccines present promising novel avenues for the application of DCs in glioma therapy. Although sequential administration of poly(I:C) and CpG ODN demonstrated promising efficacy in improving the prognosis of tumor-bearing mice in preclinical studies, significant attention must be paid to the clinical safety profile of these Toll-like receptor (TLR) agonists. The systemic toxicity risk associated with poly(I:C) and CpG ODN primarily stems from their potential to induce excessive immune activation. In severe cases, this may lead to systemic inflammatory response or cytokine release syndrome, particularly when administered intravenously or at high doses. While adoptive T cell therapy may partially mitigate these risks, researchers should remain cautious of these limitations during clinical translation. In terms of mechanistic insights, classical oncology literature posits that tumor cells can upregulate the expression of PD-L1 through transcription factors such as ATF3, thereby exhausting effector T-cell function and achieving immune evasion [25,26]. This apparent contradiction may be attributed to the divergent roles of ATF3 in tumor cells versus dendritic cells. Furthermore, in the present study, dendritic cells were not chronically exposed to the complex tumor microenvironment, which could also explain why ATF3 exhibited antitumor effects in our experimental setting. Based on these findings, future research directions emerging from this study include utilizing the immortal nature of engineered DCs to construct cellular therapies capable of the sustained autonomous expression of immune-stimulatory molecules (e.g., cytokines, co-stimulatory ligands) and employing nanoparticle-based delivery systems for the controlled release or targeted delivery of TLR agonists or other small-molecule immunomodulators within DCs. Such strategies hold the potential to overcome the temporal and operational complexities associated with sequential administration, paving the way for the development of a new generation of highly efficient and intelligent DC vaccines.

## 5. Conclusions

The primary objective of this study was to explore a novel strategy to enhance the efficacy of dendritic cell vaccines. Our in vitro investigations revealed that the combined application of poly(I:C) and CpG ODN synergistically upregulated the expression of key stimulatory molecules, such as CD86 and MHC-I, on DCs. Furthermore, this combination elicited a synergistic enhancement in IL-12p70 secretion compared to either agent used alone. Beyond phenotypic changes, DCs treated with the combination exhibited superior migratory capacity and a heightened ability to stimulate the proliferation and perforin expression of CD8^+^ T cells. These functional improvements strongly suggested that the combined regimen could confer superior anti-tumor efficacy to the DC vaccine. In vivo experiments confirmed this hypothesis. Antigen-loaded DCs activated with the combination significantly reduced tumor volume and prolonged the survival of tumor-bearing mice. To elucidate the underlying mechanism, we conducted transcriptome sequencing on DCs subjected to the different treatments. Integrated bioinformatics analysis led us to hypothesize that TLR9 activation by CpG ODN might promote the upregulation of TLR3 expression. Subsequent validation via Western blot and qPCR confirmed that the synergistic effect is mediated through the p38 MAPK-ATF3 signaling axis, which links TLR9 activation to enhanced TLR3 expression. Given that this receptor upregulation is a time-dependent process, we rationally designed a sequential administration protocol. This optimized approach demonstrated even greater efficacy, further suppressing tumor growth and extending survival in two distinct glioma models compared to simultaneous combination therapy. Preliminary experiments using engineered human DCs indicated that sequential stimulation might enhance CD8^+^ T cell proliferative capacity even at lower total stimulus doses, hinting at its potential clinical translatability. In summary, this study identifies a feasible and cost-effective method for enhancing DC vaccines. The primary significance lies in uncovering a novel mechanistic link between TLR9 and TLR3 signaling via the p38 MAPK-ATF3 axis, and in proposing an optimized sequential administration protocol that demonstrates promising clinical translation value in preliminary assessments. However, this study has limitations that warrant further investigation. Mechanistically, the precise sequences bound by ATF3 to upregulate TLR3 expression remain undefined. In vivo, the specific contributions of CD4^+^ and CD8^+^ T cells to the formation of anti-tumor memory T cells were not fully delineated. Furthermore, practical constraints prevented us from using adoptive T-cell transfer models to more robustly evaluate the clinical potential of this approach with human DCs. Despite the long path ahead to clinical application, and given the continuous emergence of new immunotherapies, optimized DC vaccines may one day play a pivotal role in combating glioma.

## Data Availability

The original contributions presented in the study are included in the article, further inquiries can be directed to the corresponding authors.
